# Automated detection and classification of early AMD biomarkers using deep learning

**DOI:** 10.1038/s41598-019-47390-3

**Published:** 2019-07-29

**Authors:** Sajib Saha, Marco Nassisi, Mo Wang, Sophiana Lindenberg, Yogi kanagasingam, Srinivas Sadda, Zhihong Jewel Hu

**Affiliations:** 1 Doheny Eye Institute, Los Angeles, CA 90033 USA; 2 Dept. of Ophthalmology, David Geffen School of Medicine, The University of California, Los Angeles, CA 90033 USA; 3 Australian e-Health Research Centre, CSIRO, Perth, Australia

**Keywords:** Predictive markers, Macular degeneration

## Abstract

Age-related macular degeneration (AMD) affects millions of people and is a leading cause of blindness throughout the world. Ideally, affected individuals would be identified at an early stage before late sequelae such as outer retinal atrophy or exudative neovascular membranes develop, which could produce irreversible visual loss. Early identification could allow patients to be staged and appropriate monitoring intervals to be established. Accurate staging of earlier AMD stages could also facilitate the development of new preventative therapeutics. However, accurate and precise staging of AMD, particularly using newer optical coherence tomography (OCT)-based biomarkers may be time-intensive and requires expert training which may not feasible in many circumstances, particularly in screening settings. In this work we develop deep learning method for automated detection and classification of early AMD OCT biomarker. Deep convolution neural networks (CNN) were explicitly trained for performing automated detection and classification of hyperreflective foci, hyporeflective foci within the drusen, and subretinal drusenoid deposits from OCT B-scans. Numerous experiments were conducted to evaluate the performance of several state-of-the-art CNNs and different transfer learning protocols on an image dataset containing approximately 20000 OCT B-scans from 153 patients. An overall accuracy of 87% for identifying the presence of early AMD biomarkers was achieved.

## Introduction

Age-related macular degeneration (AMD) is the leading cause of blindness among elderly individuals in the developed world. AMD affects 1 in 7 over the age of 50, with the incidence increasing with age^1^. It is estimated that about 8 million people in the United States who are 55 and above years old have monocular or binocular intermediate AMD or monocular advanced AMD^2^. Advanced AMD is defined by the presence of central atrophy or macular neovascularization and is commonly associated with visual loss. The chances of progression to advanced AMD in 5 years period is 27% for patients with intermediate AMD^3^. For patients with already advanced AMD in the fellow eye, this chance can be as high as 43%^3^. Though treatments are now exist for patients with neovascular AMD (choroidal neovascularization, CNV), these patients likely to develop atrophy over time and appear to lose vision eventually. No proven treatment is currently available in the setting of non-neovascular disease to prevent the progression of atrophy, termed geographic atrophy (GA). Some agents under study may slow the progression of GA^4^, however, it is desirable to intervene in AMD patients at an earlier stage, prior to development of irreversible atrophic changes or destructive exudation from CNV. In conducting such early intervention studies, it is critical to identify those patients with high risk for progression to advanced AMD.

Historically, color fundus photograph has been the gold standard for determining early AMD. Relying on color fundus photographs, various studies^3,5,6^ have identified risk factors for progression that include the manifestation of large drusen, an increased total drusen area, hyperpigmentation, and depigmentation. Based on these risk factors, the Age-Related Eye Disease Study (AREDS) defined a nine-step detailed scale^7^, as well as a simplified scale^8^ for assessing the risk of progression of AMD. The simple scale used only two factors namely large drusen and pigmentary changes to assess the eye and was designed for easy clinical application. Optical coherence tomography (OCT) has largely supplanted color fundus photography in clinical practice in recent days, because OCT provides three-dimensional cross-sectional anatomic information of retinal abnormalities, which color fundus photography cannot provide. Several novel OCT-based features have been identified by a number of studies to signal risk of AMD progression^9^. Higher central drusen volume^10^, intraretinal hyperreflective foci^11^, heterogeneous internal reflectivity within drusenoid lesions (IRDL)^12^, and reticular pseudodrusen or subretinal drusenoid deposits (SDD)^13–15^, are some of the promising ones that appear to signal risk for progression to advanced AMD^9^. OCT provides excellent opportunities to better understand AMD and its associated biomarkers, however, it generates massive image data volume (up to hundreds of B-scans per examination), which makes manual analysis of OCT extensively time-taking and impractical in many circumstances.

A number of approaches have already been proposed using retinal OCT images for automated and semiautomated analysis of AMD biomarkers, namely drusen^16–22^, GA^23–26^, pigment epithelial detachment (PED)^27–30^, and intra-/sub-retinal fluid^31–33^. Algorithms for drusen detection and segmentation^23–26^, primarily depend on on the difference between the actual retinal pigment epithelial (RPE) surface and a calculated ideal RPE or Brunch’s membrane for automated recognition of drusen. In contrast to other methods, de Sisternes *et al*.^19^ utilized 11 drusen specific features for determining the likelihood of progression from early and intermediate AMD to exudative AMD. Information about drusen texture, its geometry, reflectivity, number, area as well as volume were used for computing the likelihood. GA detection algorithms^27–30^ on OCT mainly used a partial summed voxel projection (SVP) of the choroid relying on the increase in reflectance intensity beneath Bruch’s membrane in the GA. Chen *et al*.^23^ proposed a classic method in this category. The method first segmented the RPE. A partial SVP underneath the RPE was subsequently generated and the en face image was computed using the average axial intensity within the slab. Finally, GA was identified GA with the help of an active contour model and using the en face projection. Chiu *et al*.^34^ used abnormal thinning and thickening of the RPE-drusen complex (RPEDC), defined by the inner aspect of the RPE plus drusen material and the outer aspect of Bruch’s membrane, to identify GA and drusen, respectively. In order to quantify PED volume in OCT, Ahlers *et al*.^27^ and Penha *et al*.^28^ relied on a similar principle as for drusen detection based on comparing the actual RPE position with the ideal or normal RPE position. To quantify PED on OCT, graph-based surface segmentation was used by Sun *et al*.^29^ and Shi *et al*.^30^. Algorithms for intra- and subretinal fluid detection in OCT relied on a number of image analysis techniques such as gray level^31^, gradient-based segmentation^32^, active contours^33^, and convolutional neural networks^35^. Schmidt-Erfurth *et al*.^36^ proposed a method for predicting individual disease progression in AMD relying onmachine learning and other advanced image processing techniques. Imaging data that include segmented outer neurosensory layers and RPE, drusen and hyperreflective foci, together with demographic and genetic input features were used for the prediction. The method predicted the risk of conversion to advanced AMD, with area under curve (AUC) of 0.68 and 0.80, respectively for CNV and GA. An overview and summary regarding various methods for automated analysis of AMD biomarkers on optical coherence tomography has recently been published by Wintergerst 2017^35^.

**Figure 1:**
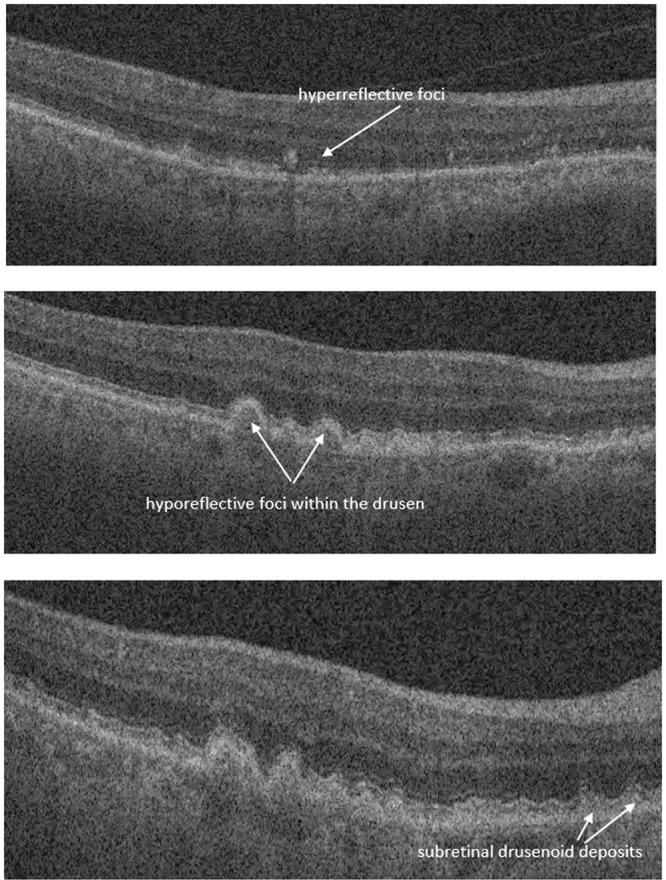
Example of hyperreflective foci, hyporeflective foci within drusen and subretinal drusenoid deposit.

In our study, we report on the performance of an automated method for detection and classification of multiple early AMD biomarkers: namely, reticular pseudodrusen, intraretinal hyperreflective and hypoflective foci (Fig. 1). Worth mentioning, the proposed study has been inspired by the results of our group^9^ that found a great association of reticular pseudodrusen, intraretinal hyperreflective and hypoflective foci, and drusen volume with overall AMD progression. Drusen volume was the least predictive among these four biomarkers. In addition to that todays machines are already capable to perform drusen volume measurements. That is why, this paper mainly focuses on developing artificial intelligent methods for the assessment of SSD, HRF and hRF.

In clinical practice, this tool could be employed as a screening method to rapidly identify B-scans which require further attention and critical analysis by the practitioner, thus increasing the accuracy and efficiency of diagnosis.

## Methods

### Dataset

Spectral domain (SD)-OCT images of 153 patients who were diagnosed with early or intermediate AMD in at least one eye at the Doheny Eye Centers between 2010 and 2014, were collected and analyzed for this study. All eyes were captured using a Cirrus HD-OCT camera (Carl Zeiss Meditec, Dublin, CA) with 1024 (depth) 512 × 128 cube (2 × 6 × 6 mm) centered on the fovea. All images were de-identified according to Health and Insurance Portability and Accountability Act Safe Harbor prior to analysis. Ethics review and institutional review board approval from the University of California – Los Angeles were obtained. The research was performed in accordance with relevant guidelines/regulations, and informed consent was obtained from all participants. A total of 19584 OCT B-scans were available for this study and about 90% of these B-scans did not contain features of disease (i.e. were normal). In order to balance the number of disease and normal B-scans, only a portion of the normal images were used for our experiment, and concurrently data augmentation was performed for the disease cohort.

All B-scans were graded by certified expert Doheny Image Reading Center (DIRC) OCT graders. B-scans were classified as having a disease feature present only if the grader was >90% confident that the feature was present. A total of 1050 OCT B-scans were graded as having definite subretinal drusenoid deposit, 326 B-scans had definite intraretinal hyper-reflective foci, and 206 B-scans had definite hyporeflective drusen. In addition, subretinal drusenoid deposits, intraretinal hyperreflective foci, and hyporeflective drusen were graded to be questionably present (i.e. grading confidence of 50–90%) in 308, 85, and 45 B-scans, respectively. As these questionable B-scans had some level of ambiguity, they were excluded from the experiment. In order to avoid any bias in training the deep CNN, we decided to use about same number of images both for the disease and no-disease category. We performed data augmentation specifically by rotation (in the range of −5 to 5 degrees), shearing (in the range 0.2), scaling (in the range 0.2) and flipping, to increase the number of diseased images by 10~15 times. Table 1 summarizes the number of B-scans used for this experiment. 90% of these B-scans were used for training and 10% were used for testing. Training and test images were selected randomly. Further to that, training set and test set were divided prior to doing any data augmentation, to avoid data impurity.

**Table 1:** Summary of the number of B-scans used for the experiment.

Early AMD pathologies	No of B-scans initially available		No of B-scans used for the experiment	
	Disease	No-disease	Disease^†^	No-disease^Γ^
SDD	1050	18222	10500	10800
HRF	326	19173	4890	5300
hRF	206	19933	3090	3100

^**†**^Following augmentation.

^Γ^Selected randomly.

### Grading protocol for OCT B-scans

Each of the B-scans of the 512 × 128 macular cube was individually assessed to determine the presence of intraretinal hyperreflective foci (IHRF), hyporeflective foci (hRF) within druseniod lesion (DL) and subretinal drusenoid deposit (SDD)^9^. Drusenoid lesions typically appear homogeneous internally with a ground-glass medium reflectivity^9^. Graders explicitly looked for the occurrence of hyporeflective foci within the drusen (Fig. 1). Knowing the requirement of the presence of enough number of pixels inside a drusen to reliably determine hRF, drusenoid lesions with a height of at least 40 μm was only taken into account while assessing the internal reflectivity^37^. IHRFs were defined as discrete, well-circumscribed hyperreflective lesions within the neurosensory retina, and a reflectivity at least as bright as the RPE band (Fig. 1)^38^. A minimum size of 3 pixels was set for IHRFs, to differentiate from noise and retinal capillaries. SDDs were defined as medium-reflective hyper-reflective mounds or cones, either at the level of the ellipsoid zone or between the ellipsoid zone and the RPE surface (Fig. 1)^9^. A lesion was considered present if the grader had greater than 90% confidence that it was present in at least one B-scan, which is the conventional practice of the reading-center^9^.

### Identifying early AMD biomarkers using deep learning

We used deep learning^39^ for automated identification of these OCT-based AMD biomarkers. Deep learning, also known as deep structured learning or deep machine learning, is the process of training a neural network to perform a given task^40^. In comparison to traditional machine learning approaches that still depend on hand-crafted features to extract valuable information from data, deep learning employs machine to learn the features by itself^41^. Thus, deep learning approach is more objective and robust. In addition to that, traditional machine learning approaches require manual outlining of pathology/features, which is expensive and time consuming to produce;^42^ whereas, deep learning requires only the label of the data, which can be produced quickly. Importantly enough, in recent years, deep learning techniques are found to beat traditional machine learning approaches with significant margins and have become state-of-the-art in image classification, segmentation, and object detection in medical and ophthalmic images^42^. A problem of deep learning though, was the requirement of huge labelled data; however, through ‘transfer learning’^43^, now it is possible to overcome this requirement. Hence, deep learning coined with transfer learning is an ideal fit in the context.

**Figure 2:**
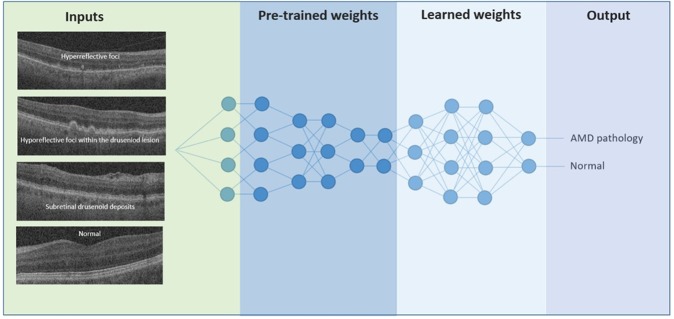
Deep learning for identifying the presence of early AMD biomarkers. Neuron connections shown here are for illustration only. Inspired by the schematic representation of Kermany *et al*.^51^.

Deep convolution neural networks (CNNs) that were specially designed to process images were trained from the intensities of the OCT B-scans. During the training process we initialized the parameters of the CNN using transfer learning, as shown in Fig. 2. More specifically, we used pre-trained models that were already trained using a very large image dataset named ImageNet^44^ to initialize the network parameters, which were then fine-tuned using the provided image dataset. Transfer learning enables fast network training with less epochs, thus further avoids over fitting and ensures robust performance. It is a promising alternative to full training and is already applied in many areas of biomedical imaging including retinal imaging^41,43^.

While fine tuning the CNN, we considered 11 different setups - fine-tuning the last 0%, 10%, 20%, 30%, 40%, 50%, 60%, 70%, 80%, 90% and 100% layers. For a CNN with *L* layers, if *α*_*l*_ denote the learning rate of the *l*-th layer in the network, 0% fine-tuning or fine-tuning only the last layer of the network was defined as setting *α*_*l*_ = 0 for *l* ≠ *L*. Likewise for *P*% fine-tuning we train up-until *P* + 1 layers.

Worth mentioning, typically, the initial layers of CNN learn low-level image features. In general, these low-level features do not vary significantly from application to application. The top layers of CNN learn high-level features, which are specific to the application at hand. Therefore, fine-tuning only the top few layers is usually sufficient while training CNN^43^. However, when source and target applications differ substantially, fine-tuning only the last few layers may not be sufficient. Therefore, an efficient fine-tuning strategy is to start from the last layer and then incrementally add more layers in the update process until the desired performance is reached.

For each of the pathology types we trained three different nets namely Inception-v3^45^, ResNet50^46^, and InceptionresNet50^47^. For each net, we conducted the experiment on 11 different setups (e.g. fine-tuning strategy) as explained in ‘Identifying early AMD biomarkers using deep learning’. We compared the performance of different setups for all three CNNs. Experiments were conducted likewise for each of the three AMD biomarkers.

### Automated segmentation of retinal layer using ReLayNet

Prior to feeding the image into CNN for pathology detection and classification, we performed a pre-segmentation of the retinal layers using ReLayNet^48^, as our early AMD biomarkers tend to be localized to specific retinal layers. SDD usually appear above the inner RPE surface, hyporeflective drusen are usually located above the Bruch’s membrane/inner choroid surface, and hyperreflective foci may appear in several different outer retinal layers. ReLayNet produced an 8-layer segmentation mask, which were then used to compute a binary mask that only contains the retinal region spanning from the outer nuclear layer (ONL) to Bruch’s membrane/inner choroid. It is worth noting that ReLayNet itself is a deep learning framework which is specially designed to perform segmentation of retinal layers in OCT B-scans. The framework is validated on a publicly available benchmark dataset with comparisons against five state-of-the-art segmentation methods including two deep learning based approaches to substantiate its effectiveness. The computed binary mask is finally imposed on the input image to define the region of interest.

**Figure 3:**
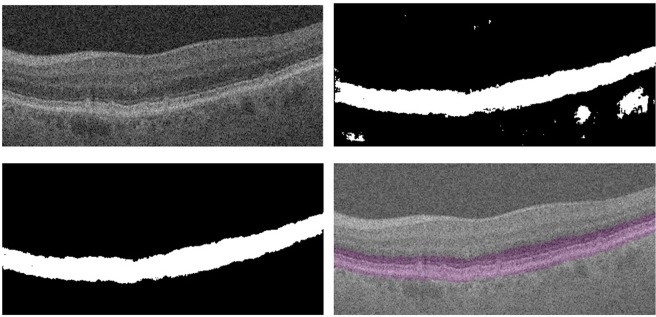
Top-left: an example OCT B scan, top-right: region of interest mask generated based on ReLayNet, bottom-left: region of interest mask generated using ReLayNet and other image pre-processing technique, bottom-right: mask (shown in purple) superimposed on the image.

The pixel level segmentation purely based on ReLayNet contained some outliers that includes small holes within the region of interest, and scattered group of pixels/small regions. We performed morphological operations including region filling and length based object removal to avoid those outliers. Figure 3 shows an example OCT B-scan, and corresponding region of interest mask generated purely based on ReLayNet and ReLayNet with other pre-processing.

### Performance metrics

The performance metrics which we used included accuracy, sensitivity, specificity and area under the curve (AUC). Accuracy was defined as the ratio of the number of correct identifications made over the total number of images available on the validation set. Sensitivity was defined as the proportion of actual positives that are correctly identified, whereas specificity was defined as the proportion of actual negatives that are correctly identified. To compare different setups and nets, and receiver operating characteristic (ROC) curves were mainly used. ROC curves plot the detection probability (i.e. sensitivity) versus false alarm rate (i.e. 1-specificity).

### Use of human participants

Ethics review and institutional review board approval from the University of California – Los Angeles were obtained. The research was performed in accordance with relevant guidelines/regulations, and informed consent was obtained from all participants.

## Results

**Figure 4:**
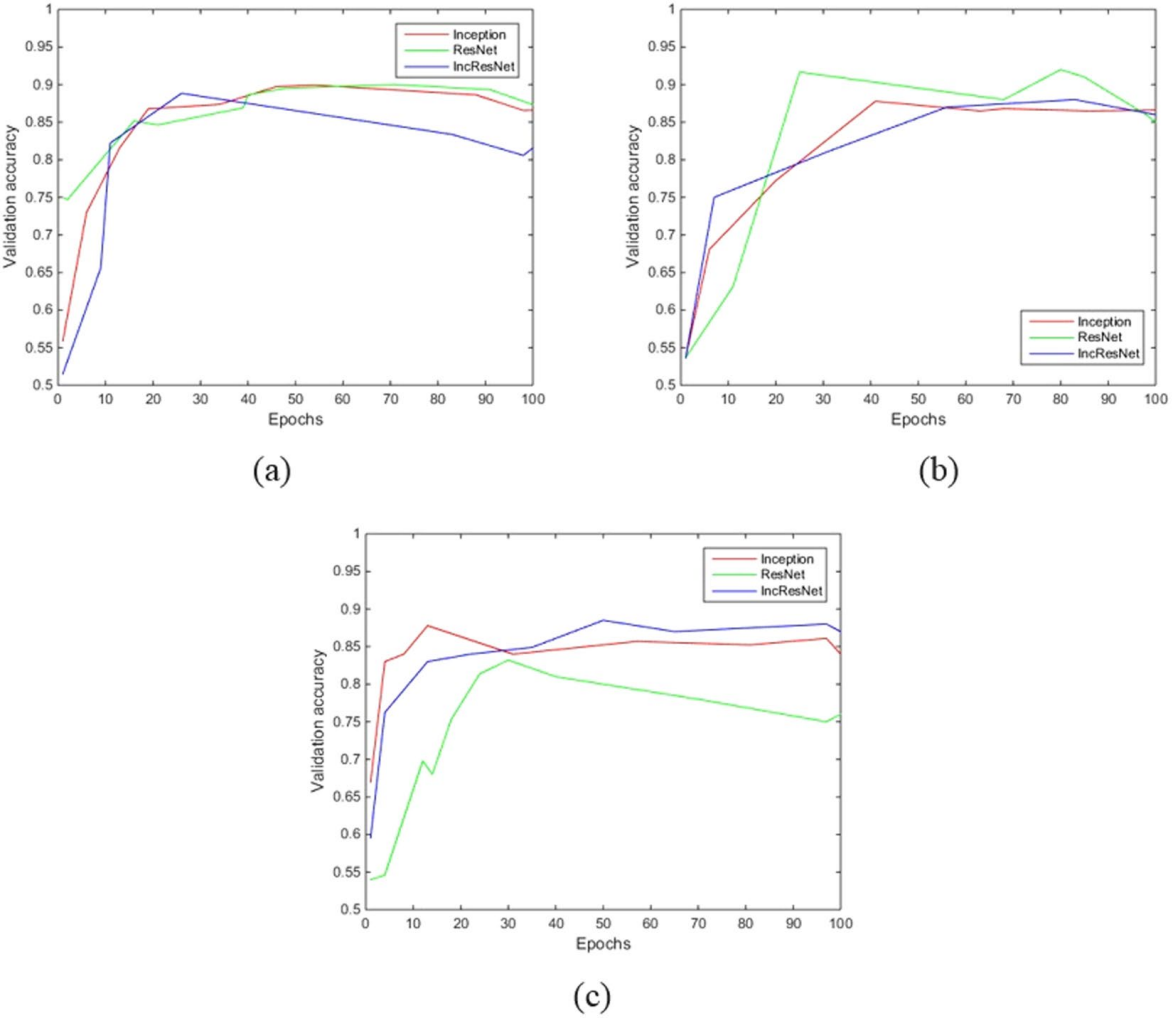
Fitted curve representing the validation accuracy over epochs by the three different nets for identifying the presence of (**a**), IHRF (**b**) hRF, and (**c**) SDD.

Figure 4 shows the fitted curve representing the validation accuracy over epochs by the three different nets for identifying the presence early AMD pathologies. The validation accuracy (against epochs) of the best setups are only shown.

**Figure 5:**
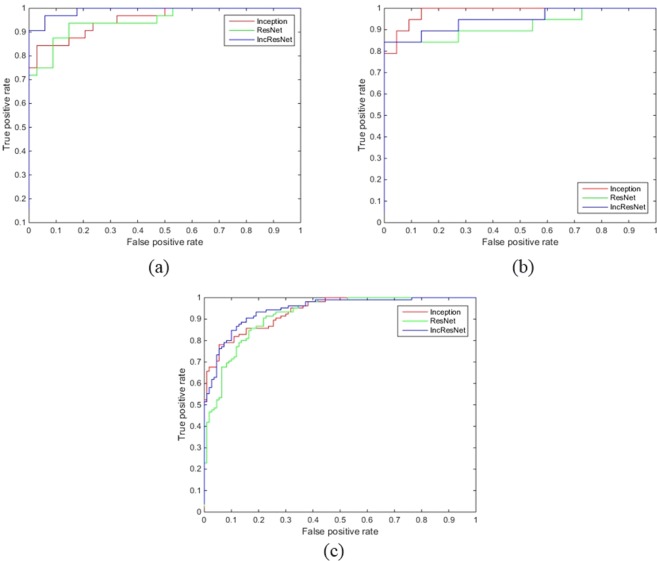
Receiver operating characteristic (ROC) curve of the three different nets for identifying the presence of (**a**) IHRF, (**b**) hRF, and (**c**) SDD.

From Fig. 4 it can be inferred that accuracy improvement gets saturated by 10~50 epochs for SDD, about 20~40 epochs for IHRF, and about 20~40 epochs for hRF. The performance between different nets are not to an extent that would be relevant in practice. However, from the receiver operating characteristic (ROC) curves as shown in Fig. 5, InceptionResNet is better suited for detecting the presence of SDD and IHRF; and Inception is better suited for identifying the presence of hRF. Table 2 summarizes the sensitivity, specificity, AUC and accuracy obtained by different models.

**Table 2:** Sensitivity, specificity, AUC and accuracy obtained by different models.

	Sensitivity	Specificity	AUC	Accuracy
**IHRF (%)**				
Inception-v3	81	97	95	89
ResNet50	87	91	95	89
InceptionResNet50	78	100	99	89
**hRF (%)**				
Inception-v3	79	95	98	88
ResNet50	74	100	91	88
InceptionResNet50	84	90	94	88
**SDD (%)**				
Inception-v3	83	85	92	84
ResNet50	96	65	91	80
InceptionResNet50	79	92	94	86

In aggregate, experiments on all the three CNNs show promising results on identifying the presence of early AMD pathologies. Accuracy ranged from 86~89%. SDD can be identified with an accuracy of 80%~86%. Accuracy for identifying the presence of IHRF and hRF were 89% and 88%, respectively. SDD can be best detected by InceptionResNet, having sensitivity, specificity and accuracy of 79%, 92% and 86%, respectively. HRF was also best detected by InceptionResNet with sensitivity and specificity of 78%, and 100%, respectively. hRF was best detected by Inception with sensitivity and specificity of 79% and 95%, respectively.

## Discussion

We propose an automated system for identifying the presence of early AMD biomarkers from OCT B-scans. By employing transfer learning algorithm, the proposed system showed good performance for this application without the need for a highly specialized deep learning machine or a database of millions of images. The system provides numerous benefits, including consistency in prediction (because a machine will make same prediction for same image each time) and instantaneous reporting of results. In addition to that since the algorithm can have multiple operating points, its sensitivity and specificity can be adjusted to meet specific clinical requirements, for example high sensitivity for a screening application.

One fundamental limitation of deep learning were the requirement of huge number of training images. However, with the development of transfer learning paradigm, this is not a limitation any more. Relying on transfer learning state-of-the-art classification performance is achieved using only couple of hundreds to thousands of images^49,50^. A very relevant example is the recent study made by Christopher *et al*.^50^. Christopher *et al*. have used a fundus dataset of 14,822 images and relying on transfer learning, they have achieved state-of-the-art accuracy of 91% in distinguishing glaucomatous optic neuropathy eyes from healthy eyes. Our study that involved about 20,0000 OCT scans and used transfer learning is fully sufficient and the results are representative.

Although we are able to train a highly accurate deep learning model here, with a relatively small training dataset, unsurprising, it’s performance would be inferior to that of a model which is trained using ‘full training’, or in other words from a random initialization on an extremely large dataset of OCT images. All the network weights of the model are directly optimized when full training is performed. However, OCT images in such a volume to train a blank CNN are difficult to ascertain.

Similar to other transfer learning based models, the performance of our system depends highly on the weights of the pre-trained model. Therefore, the performance of the system would likely be enhances when more advanced pre-trained models that are trained with even larger dataset are used. In this work we used the pre-trained models that were trained on the ImageNet dataset which is biggest dataset to our knowledge for such classification.

The system performs a pre-segmentation of the region of interest prior to sending the images to CNN, in an aim to eliminate pathologic features that are present outside of the region spanning from the ONL to Bruch’s membrane/inner choroid. Theoretically, it should increase the performance of the system. However, we did not observe any significant improvement that would be relevant in practice. In a post-hoc review of images in our dataset, we found that the B-scans, which had AMD-related pathologic features outside of the ONL to Bruch’s membrane/inner choroid region, also had AMD features present within the region of interest. This likely explains why we did not observe any significant improvement.

Since, the number of diseased images in our dataset were significantly less than the number of normal images, to ensure fair learning of the CNNs we considered two different arrangements during training. In the first arrangement all the diseased images were considered, whereas normal images are chosen randomly to match the number of images in the diseased category. Data augmentation was performed for each of the categories. In the second arrangement we performed data augmentation of the diseased images, and randomly chose similar number (after augmentation) of normal images as explained in section the Dataset section. Unsurprising, the accuracy for classification of arrangement −1 (summarized in Table 3) was less than the classification accuracy of arrangement −2, which meant data augmentation is not fully able to generate all the different scenarios that we observe naturally. That explains why arrangement −2 was considered in implementing the system.

**Table 3:** Accuracy obtained by different models.

CNNs	Accuracy (%)		
	IHRF	hRF	SDD
Inception-v3	89	88	84
ResNet50	88	87	81
InceptionResNet50	89	87	85

There are limitations to our system. One important limitation ascends by the nature of deep neural networks, in which the network was provided with only the image and associated label, without explicit definitions of features (e.g. SDD, IHRF or hRF). Because the network “learned” the features that were most critical for correct classification, there is a chance that the algorithm is using features previously not recognized or ignored by humans. Another limitation is that the study used images collected from a single clinical site.

## Conclusions

In this study, we sought to develop an effective deep learning method to identify the presence of early AMD biomarkers from OCT images of the retina. We compared the performance of several deep learning networks in an aim to identify the best net in this context. We also incorporated several image pre-processing techniques to improve the classification accuracy. We obtained an accuracy of 86% to identify the presence of subretinal drusenoid deposit. Intraretinal hyperreflective foci and hyporeflective foci within drusen were detected with an accuracy of 89% and 88%, respectively. Worth mentioning, the rate of disagreement between different graders are above 20%^9,50^. An automated system that achieves an accuracy of 86~89% with the gold standard, and produces classification with a fraction of time required by an expert grader, is a promising choice to move forward.

We used 90% of the data for training to ensure the robustness of the algorithm. The results from the 10% testing data has indicated a good performance. Our clinic is continuously collecting data with new patients and we will further test our algorithm with future new data.

Given the increasing burden of AMD on the healthcare system, the proposed automated system is highly likely to perform a vital role in decision support systems for patient management and in population and primary care-based screening approaches for AMD. With the growing and critical role of OCT in the understanding and monitoring of AMD progression, the proposed automated system should be of clinical value, not only for increasing diagnostic accuracy and efficiency in clinical practice, but also in the design and execution of future early intervention therapeutic trials.

## Data Availability

The code generated during the study is accessible from the corresponding author based on reasonable request and subject to the rule/regulatory of the institute.
